# Potential of extravasated platelet aggregation as a surrogate marker for overall survival in patients with advanced gastric cancer treated with preoperative docetaxel, cisplatin and S-1: a retrospective observational study

**DOI:** 10.1186/s12885-017-3279-4

**Published:** 2017-04-27

**Authors:** Hiroto Saito, Sachio Fushida, Tomoharu Miyashita, Katsunobu Oyama, Takahisa Yamaguchi, Tomoya Tsukada, Jun Kinoshita, Hidehiro Tajima, Itasu Ninomiya, Tetsuo Ohta

**Affiliations:** Department of Gastroenterological Surgery, Division of Cancer Medicine, Graduate School of Medical Science, Kanazawa University, 13-1 Takara-machi, Kanazawa, Ishikawa 920-8641 Japan

**Keywords:** Gastric cancer, Platelets, Preoperative chemotherapy, Chemoresistance, Surrogate marker

## Abstract

**Background:**

The theory of extravasated platelet aggregation in cancer lesions was recently introduced. We investigated the association of platelet aggregation in gastric cancer stroma with clinicopathological features, chemotherapeutic response, pathological response, and survival.

**Methods:**

The study comprised 78 patients with advanced gastric cancer who had undergone gastrectomy with or without combination of docetaxel, cisplatin and S-1 (DCS) as preoperative chemotherapy between 2005 and 2014. The patients were divided into two groups: patients who had received preoperative DCS therapy forming the p-DCS group and patients who had not received preoperative DCS therapy forming the control group. The 39 patients in the control group had received gastrectomy and postoperative chemotherapy of S-1 alone. Platelet aggregation in biopsy specimens before preoperative DCS therapy in the p-DCS group and at the time of diagnosis in the control group were evaluated using CD42b immunohistochemical staining.

**Results:**

Twenty-four patients in the p-DCS group and 19 in the control group were found to have platelet aggregation in their cancer stroma. Patients with histologically confirmed platelet aggregation had significantly higher rates of chemoresistance (58.3%) than those without platelet aggregation (20.0%) (*P* = 0.019). According to multivariate analysis, CD42b expression (odds ratio: 5.102, 95% confidence interval: 1.039–25.00, *P* = 0.045) was correlated with chemoresistance. CD42b expression and histological non-responder status were both significantly correlated with poor overall survival (OS) (*P* = 0.012, *P* = 0.016); however, RECIST was not correlated with OS. In the control group, CD42b expression was also significantly correlated with poor overall survival (OS) (*P* = 0.033). In the p-DCS group, according to multivariate analysis, male sex (hazard ratio: 0.281, 95% confidence interval: 0.093–0.846, *P* = 0.024) was correlated with good prognosis and CD42b expression (hazard ratio: 4.406, 95% confidence interval: 1.325–14.65, *P* = 0.016) with poor prognosis.

**Conclusions:**

This study suggests that platelets in gastric cancer stroma may create a favorable microenvironment for chemoresistance. CD42b immunohistochemical staining of biopsy specimens is a promising candidate for being a prognostic marker in patients with gastric cancer.

## Background

An estimated 951,600 new cases of gastric cancer and 723,100 deaths occurred in 2012 [1]. Although the incidence of gastric cancer has decreased in recent decades, it remains one of the leading causes of cancer-related death in East Asia. S-1 is an effective postoperative chemotherapy for East Asian patients who have undergone a D2 dissection for locally advanced gastric cancer [2]. Multimodality treatment, including chemotherapy and surgery, has reduced gastric cancer mortality and improved quality of life. Some studies [3–7] have suggested that preoperative chemotherapy followed by surgery is improves long-term prognosis of advanced gastric cancer. However, there are no established biomarkers for screening the efficacy of preoperative or postoperative chemotherapy.

Two methods are currently available for evaluating tumor responses to chemotherapy. The Response Evaluation Criteria in Solid Tumors (RECIST) [8] have been widely used to evaluate tumor responses. However, RECIST cannot always be used in the preoperative setting because there may be no measurable lesions in patients with resectable gastric cancer. In contrast, histological evaluation of the primary tumors is commonly used after surgery for the patients treated with preoperative chemotherapy. Some studies have reported that histological evaluation yields more valid response criteria of preoperative treatment than RECIST [9, 10].

Platelets are primarily recognized as key regulators of thrombosis and hemostasis. Bambace and Holmes [11] have reported that platelets are linked to key steps in cancer progression and metastasis. After tumor cells migrate into the bloodstream, they induce platelet aggregation and the platelet-coating protects tumor cells from immune surveillance and shear stress. Platelets also facilitate cancer cell adherence to vascular endothelial cells, which leads to extravasation into the stroma and formation of secondary tumors [12]. However, there are few reports regarding the role of platelets in primary tumors. Qi et al. [13] reported that platelet aggregation within colorectal cancers is associated with tumor stage and lymph node metastasis. Mikami et al. [14] showed that interactions between platelets and gastric cancer cells increase tumor proliferation.

A theory of extravasated platelet aggregation (EPA) in primary cancer lesions was recently introduced [15]. Several studies have focused attention on the central role of platelet interaction with cancer cells and the immune system in promoting tumor progression and distant spread through release of growth factors such as transforming growth factor (TGF)-β, vascular endothelial growth factor A, and platelet-derived growth factor into the microenvironment [15]. TGF-β enhances epithelial–mesenchymal transition (EMT) in cancer cells [16] and EMT promotes invasiveness, metastasis, and chemoresistance [17].

To clarify the presence of factors that affect chemoresistance in the cancer microenvironment, we focused on EPA in biopsy specimens from primary tumor of gastric cancer patients who treated with preoperative or postoperative chemotherapy.

## Methods

### Inclusion and exclusion criteria

Seventy-eight patients with advanced gastric cancer who had undergone gastrectomy between 2005 and 2014 were retrospectively evaluated. Thirty-nine of them had received preoperative DCS therapy (p-DCS group), whereas the remaining 39 had not received any preoperative chemotherapy (control group). The 39 patients in the control group had, however, received gastrectomy and postoperative chemotherapy of S-1 alone. Eligibility criteria were as follows: clinical Stage III and resectable Stage IV gastric cancer with fewer than three peripheral hepatic and para-aortic lymph node (PAN) metastases [18] in accordance with the Japanese Classification of Gastric Carcinoma (JCGC), 3rd English edition [19], PAN metastasis being defined as clearly enlarged (≥ 10 mm) on enhanced computed tomography (CT) scans with 2.5 mm slice thickness; absence of peritoneal metastasis on staging laparoscopy; age 20–80 years; Eastern Cooperative Oncology Group (ECOG) performance status 0 or 1; no prior chemotherapy or radiotherapy; no prior gastrectomy; no detected bleeding from primary lesion; good oral intake; and adequate hematological, hepatic, and renal function.

Patients were excluded for any of the following reasons: apparent infection; serious comorbidity such as cardiovascular disease, pulmonary fibrosis, pneumonia, bleeding tendency, uncontrolled hypertension, poorly controlled diabetes mellitus, and other serious medical conditions; synchronous or metachronous active malignancy; central nervous system disorder; history of severe drug-induced allergy; and pregnancy or breastfeeding.

### Treatment

In the p-DCS group, patients had received two cycles of preoperative chemotherapy consisting of 35 mg/m^2^ docetaxel as a 1-h intravenous infusion on days 1 and 15; 35 mg/m^2^ cisplatin as a 2-h intravenous infusion on days 1 and 15 with hyperhydration; and 40 mg/m^2^ S-1 twice daily on days 1–14 every 4 weeks. At least 4 weeks after the completion of two cycles of DCS therapy, curative gastrectomy and D2 lymphadenectomy plus PAN dissection (PAND) and hepatectomy had been performed. Lymph node dissection was performed in patients with PAN metastasis diagnosed by enhanced helical CT, which was defined as lymph node station No. 16a2 and b1 (16a2b1PAN) between the upper margin of the celiac artery and lower border of the inferior mesenteric artery [19].

In the control group, administration of S-1 was started within 6 weeks after gastrectomy and continued for 1 year. The treatment regimen consisted of 6-week cycles in which, in principle, 40 mg/m^2^ S-1 twice daily was given for 4 weeks and no chemotherapy was given for the following 2 weeks [2, 20].

### Response evaluation

After the second course of preoperative DCS therapy, the amount of tumor shrinkage was evaluated based on thin-slice helical CT and the tumor response classified into one of the following four categories in accordance with RECIST [8]: complete response (CR), disappearance of all target lesions; partial response (PR), ≥30% decrease in the combined diameters of target lesions; progressive disease (PD), ≥20% increase in the combined diameters of target lesions; and stable disease (SD), neither sufficient shrinkage to qualify for PR nor sufficient increase to qualify for PD. Patients with CR and PR were regarded as RECIST responders.

In the p-DCS group, the resected specimens were histologically evaluated, and tumor response evaluated according to the histological criteria in JCGC, 3rd English edition [19]. The histological evaluation criteria were classified into one of the following five categories according to the proportion of the tumor affected by degeneration or necrosis: grade 3, no viable tumor cells remaining; grade 2, viable tumor cells remaining in less than one-third of the tumorous area; grade 1b, viable tumor cells remaining in more than one-third but less than two-thirds of the tumorous area; grade 1a, viable tumor cells occupying more than two-thirds of the tumorous area; and grade 0, no evidence of therapeutic effect.

Ten percent or 50% residual tumor per tumor bed has been used as the cutoff percentage in Western countries, in accordance with the criteria proposed by Becker et al. [21]. In contrast, a cutoff of 33% or 67% viable tumor cells per tumor bed is commonly used in Asian countries, in accordance with the definition in JCGC, 3rd English edition [19]. Although the definition of a histological response is controversial, Kurokawa et al. [9, 10] have evaluated the results when histological responses were classified as Grade 2 or 3 and found that the results were similar to Grades 1b, 2 or 3. In this study, a histological response was defined as less than one-third of viable tumor cells (grade 2 or 3). All resected specimens were examined by the same pathologist, who assessed the extent of residual disease, disease stage, and effect of chemotherapy according to the criteria of JCGC, 3rd English edition [19].

### Immunohistochemical examination

In the p-DCS group, primary cancer lesions were biopsied by esophagogastroduodenoscopy (EGD) before commencement of preoperative chemotherapy. In the control group, biopsies were performed by EGD on diagnosis. Biopsies were taken from the edge of ulcerations associated with gastric cancer, not from the bases of such ulceration. More than five biopsy specimens were collected from each patient and evaluated immunohistochemically. Immunohistochemistry using 3-μm-thick, 10% formalin-fixed, paraffin-embedded tissue sections was performed using Dako Envision System dextran polymers conjugated to horseradish peroxidase (Dako, Carpinteria, CA, USA) to prevent any endogenous biotin contamination. The specimens were deparaffinized in xylene and rehydrated in a graded ethanol series. Endogenous peroxidase was blocked by immersing sections in 3% H_2_O_2_ in 100% methanol for 20 min at room temperature. Antigen retrieval was activated by microwaving sections at 95 °C for 10 min in 0.001 M citrate buffer (pH 7.6). After blocking the endogenous peroxidase, sections were incubated with Protein Block Serum-Free (Dako) at room temperature for 10 min to block nonspecific staining. Subsequently, sections were incubated for 2 h at room temperature with a 1:100 diluted anti-platelet antibody (anti-CD42b rabbit monoclonal, EPR6995; Abcam, Tokyo, Japan); a 1:50 diluted anti-podoplanin antibody (anti-D2–40 mouse monoclonal, Code IR072/IS072; Dako, Tokyo, Japan); a 1:50 diluted anti-forkhead box (FOX)P3 antibody (anti-FOXP3 mouse monoclonal, 236A/E7; Abcam), and a 1:50 diluted anti-SNAIL antibody (anti-SNAIL rabbit polyclonal antibody, ab180714; Abcam). Peroxidase activity was detected using 3-amino-9-ethylcarbazole enzyme substrate. Sections were incubated in Tris-buffered saline without primary antibodies as negative controls. Samples were faintly counterstained with Meyer hematoxylin.

### Evaluation of immunostaining

To evaluate the expression of CD42b in the biopsy specimens, the immunostained cells in five non-overlapping intratumoral fields were counted at 400× magnification. The average expression of CD42b was evaluated: ≥10% was defined as positive and <10% as negative [22]. In the biopsy specimens stained by D2–40, the immunostained cells were counted at 200× magnification. The percentage of podoplanin-positive (PP) cells and staining intensity (SI) were evaluated and an immunoreactivity score (IRS) calculated for each tumor as IRS = PP × SI (0 negative, 1–3 weak, 4–7 moderate, and 8–15 high). Scores were allocated as follows: 0 PP 0%, 1 PP 1%–20%, 2 PP 21%–40%, 3 PP 41%–60%, 4 PP 61%–80%, and 5 PP 81%–100%; and 0 SI negative, 1 weak, 2 moderate, and 3 strong. For IRS, ≥4 was defined as positive and <3 as negative [23].

For analysis of SNAIL, IRS was calculated by multiplication of intensity (0–3) by the percentage of stained cells (0–4). Tissue samples with scores of 0 were classified as SNAIL negative and those with scores of 1–12 as SNAIL positive [24].

To evaluate infiltration of FOXP3, five non-overlapping intratumoral fields were counted at 400× magnification and the mean number per field defined as the number of FOXP3 infiltrates for the tumor. The average number of FOXP3-positive T cells was evaluated; ≥5.5 being defined as positive and <5.5 as negative [25].

### Statistical analysis

Fisher’s exact test was used to measure the statistical significance of correlations between CD42b expression and chemotherapeutic response. Patient survival was calculated by the Kaplan–Meier method and the log-rank test was used to compare the survival rates between subgroups. Variables found to have possible associations with chemoresistance and prognosis by univariate analysis (*P* < 0.10) were subjected to multivariate analysis using multi logistic regression analysis and the Cox proportional hazards regression model, respectively. Statistical significance was set at *P* < 0.05. Data management and statistical analysis were performed using SPSS version 23 (SPSS, Chicago, IL, USA).

## Results

### Patient characteristics

From 2005 to 2014, 78 patients with advanced gastric cancer were found to be eligible, 39 of whom had received preoperative DCS therapy followed by curative gastrectomy with D2 lymphadenectomy plus PAND and/or hepatectomy (p-DCS group). The remaining 39 patients had not received preoperative DCS therapy prior to undergoing curative gastrectomy with D2 lymphadenectomy plus hepatectomy and had received postoperative chemotherapy of S-1 alone (control group). Patient characteristics are summarized in Table 1. In the p-DCS group, baseline CT showed that 16 (41%) had PAN metastases and nine (23%) hepatic metastases. The tumor stages were as follows: 13 (33%) clinical Stage III and 26 (67%) clinical Stage IV. In the control group, baseline CT showed that none had PAN metastases and one (3%) had hepatic metastases. The tumor stages were as follows: 38 (97%) clinical Stage III and one (3%) clinical Stage IV.

**Table 1:** Patient characteristics according to treatment group and response to preoperative DCS therapy evaluated by RECIST and histological evaluation criteria

Characteristic		p-DCS group	Control group
Number of patients		39	39
Age, yr.; median (range)		63.6 (30–78)	67.0 (41–80)
Gender	Male	32	25
	Female	7	14
ECOG performance status	≥1	2	0
	0	37	39
Borrmann macroscopic type	1	0	1
	2	14	10
	3	21	16
	4	1	10
	5	3	2
Differentiation	Diffuse	18	28
	Intestinal	21	11
Clinical T stage	cT0	0	0
	cT1	0	0
	cT2	5	5
	cT3	13	16
	cT4	21	18
Clinical N stage	cN0	2	0
	cN1	2	6
	cN2	21	18
	cN3	14	15
Clinical stage	0	0	0
	I	0	0
	II	0	0
	III	13	38
	IV	26	1
PAN metastasis	(+)	16	0
	(−)	23	0
Hepatic metastasis	(+)	9	1
	(−)	30	38
RECIST	CR	0	-
	PR	29	-
	SD	8	-
	PD	2	-
Histological evaluation criteria (Grade)	3	3	-
	2	19	-
	1b	4	-
	1a	11	-
	0	2	-

*CR* complete response, *DCS* docetaxel, cisplatin, and S-1, *ECOG* Eastern Cooperative Oncology Group, *PAN* para-aortic lymph node, *PD* progressive disease, *PR* partial response, *RECIST* Response Evaluation Criteria in Solid Tumors, *SD* stable disease

### Response rates

The responses to preoperative DCS therapy were assessed by RECIST and histological evaluation criteria (Table 1). The response rates were 74% with RECIST and 56% with histological criteria.

### CD42b and podoplanin expression

**Fig. 1:**
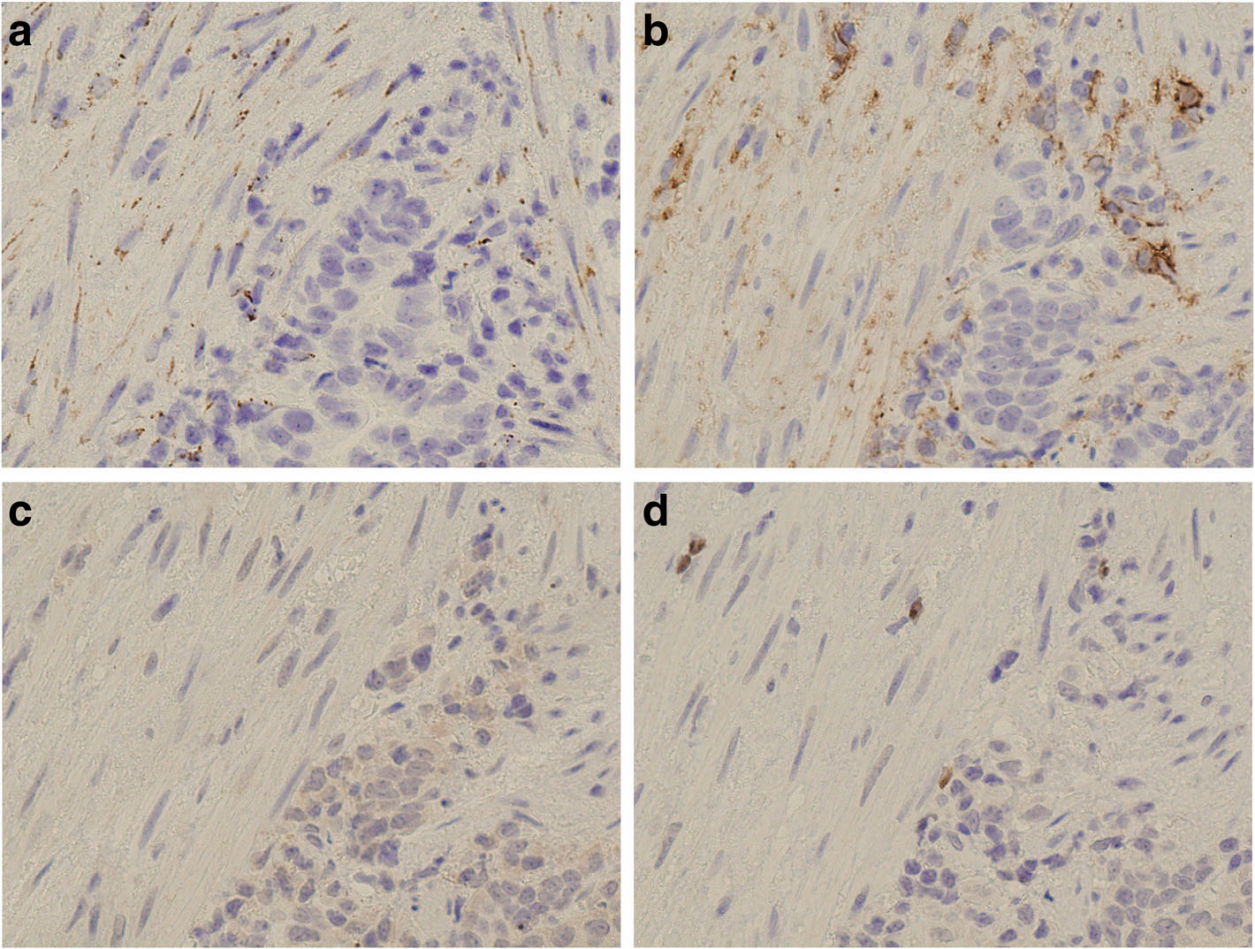
Representative photomicrograph of pretreatment biopsy specimens from advanced gastric cancer lesion. **a**: Immunohistological images of CD42b-positive platelets. Extravasated platelet aggregation (EPA) is mainly seen in the cancer stroma. Cancer-associated fibroblasts (CAFs) with platelet aggregation were observed. **b**: CAFs in gastric cancer stroma showing D2–40 expression on the membrane, whereas the cancer cells are negative for D2–40 expression. **c**: SNAIL-positivity expressed in the nuclei of cancer cells. **d**: Weak expression of forkhead box P3

In the p-DCS group, biopsy specimens were obtained from the primary gastric cancers before commencing preoperative chemotherapy. Expression of CD42b, a platelet marker, was observed around cancer-associated fibroblasts (CAFs) in the biopsy specimens (Fig. 1a) and podoplanin expression was found on the membranes of CAFs (Fig. 1b).

### Relationship between CD42b expression and histopathological variables

There were no significant associations between CD42b expression and Borrmann macroscopic type, tumor differentiation, clinical T stage, clinical N stage, PAN metastases, or hepatic metastases in either group (Tables 2 and 3).

**Table 2:** Relationship between CD42b expression and histopathological variables in the p-DCS group

Variables		CD42b (+)	CD42b (−)	*P* value
Borrmann macroscopic type	Non-type 4	24	14	0.385
	Type 4	0	1	
Differentiation	Diffuse	11	7	0.959
	Intestinal	13	8	
Clinical T stage	0	0	0	0.140
	1	0	0	
	2	5	0	
	3	7	6	
	4	12	9	
Clinical N stage	0	2	0	0.436
	1	1	1	
	2	12	9	
	3	9	5	
PAN metastasis	(+)	9	7	0.571
	(−)	15	8	
Hepatic metastasis	(+)	6	3	0.519
	(−)	18	12	

*DCS* docetaxel, cisplatin, and S-1, *PAN* para-aortic lymph node

**Table 3:** Relationship between CD42b expression and histopathological variables in the control group

Variables		CD42b (+)	CD42b (−)	*P* value
Borrmann macroscopic type	Non-type 4	4	6	0.394
	Type 4	15	14	
Differentiation	Diffuse	13	15	0.460
	Intestinal	6	5	
Clinical T stage	0	0	0	0.202
	1	0	0	
	2	5	0	
	3	5	11	
	4	9	9	
Clinical N stage	0	0	0	0.307
	1	3	3	
	2	7	11	
	3	9	6	
PAN metastasis	(+)	-	-	-
	(−)	-	-	
Hepatic metastasis	(+)	1	0	0.487
	(−)	18	20	

*DCS* docetaxel, cisplatin, and S-1, *PAN* para-aortic lymph node

In the p-DCS group, CD42b positivity was seen in 24 (62%) patients, including 10 (26%) histological responders and 14 (36%) non-responders. There were 15 (38%) CD42b-negative patients, including 12 (31%) histological responders and three (7%) non-responders. CD42b-positive patients had significantly higher rates of chemoresistance (58%) than CD42b-negative patients (20%) (*P* = 0.019).

Univariate analysis of expression of three factors (CD42b, SNAIL, and FOXP3) that are reportedly associated with chemoresistance showed significant associations between CD42b expression (*P* = 0.025) and SNAIL expression (*P* = 0.029) and chemoresistance (Table 4). These two variables were therefore considered to be potential predictors of chemoresistance and were subjected to multivariate analysis, which identified a correlation between CD42b expression and chemoresistance (odds ratio: 5.102, 95% confidence interval: 1.039–25.00, *P* = 0.045) (Table 4).

**Table 4:** Univariate/multivariate analyses of factors that are reportedly associated with chemoresistance in the p-DCS group

		Univariate analysis				Multivariate analysis		
Variable		No. of patients	OR	95% CI	*P* value	OR	95% CI	*P* value
CD42b expression	≥10%	24	5.587	(1.245–25.00)	0.025	5.102	(1.039–25.00)	0.045
	<10%	15						
SNAIL expression	(+)	30	6.993	(1.222–40.00)	0.029	6.289	(0.988–40.00)	0.052
	(−)	9						
FOXP3 expression	(+)	7	4.167	(0.696–29.94)	0.118			
	(−)	32						

*CI* confidence interval, *FOXP3* forkhead box P3, *OR* odds ratio

### SNAIL expression

In the p-DCS group, the EMT marker SNAIL was mainly expressed in the nuclei of cancer cells. Positive SNAIL expression was found in 30/39 cases (77%) (Fig. 1c); however, SNAIL expression was not correlated with CD42b expression (*P* = 0.230). There was a significant relationship between SNAIL expression and chemoresistance (*P* = 0.026) but no significant relationship between SNAIL expression and OS (*P* = 0.248).

### FOXP3 expression

In the p-DCS group, the regulatory T (Treg) cell marker FOXP3 was found in 7/39 cases (18%) (Fig. 1d). FOXP3 expression was not significantly correlated with CD42b expression (*P* = 0.686), chemoresistance (*P* = 0.205), or OS (*P* = 0.698).

### Survival curves according to chemotherapy response

**Fig. 2:**
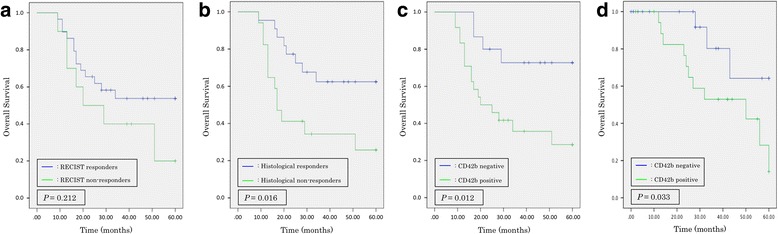
Overall survival curves for responders and non-responders in the p-DCS group and CD42b expression in the both groups. **a**: RECIST responders (*P* = 0.212; log-rank test). **b**: Histological responders (*P* = 0.016; log-rank test). **c**: CD42b expression in the p-DCS group (*P* = 0.012; log-rank test). **d**: CD42b expression in the control group (*P* = 0.033; log-rank test). *DCS,* docetaxel, cisplatin and S-1; *RECIST,* Response Evaluation Criteria in Solid Tumors

Overall survival (OS) curves for the patients in the both groups are shown in Fig. 2. In the p-DCS group, comparison of survival rates in RECIST responders and non-responders by log-rank test revealed no significant difference in prognosis (*P* = 0.212) (Fig. 2a). In contrast, OS was significantly longer in histological responders than non-responders (*P* = 0.016) (Fig. 2b) and in CD42b-negative than CD42b-positive patients (*P* = 0.012) (Fig. 2c). In the control group, the OS was significantly longer for CD42b-negative than CD42b-positive patients (*P* = 0.033) (Fig. 2d).

**Fig. 3:**
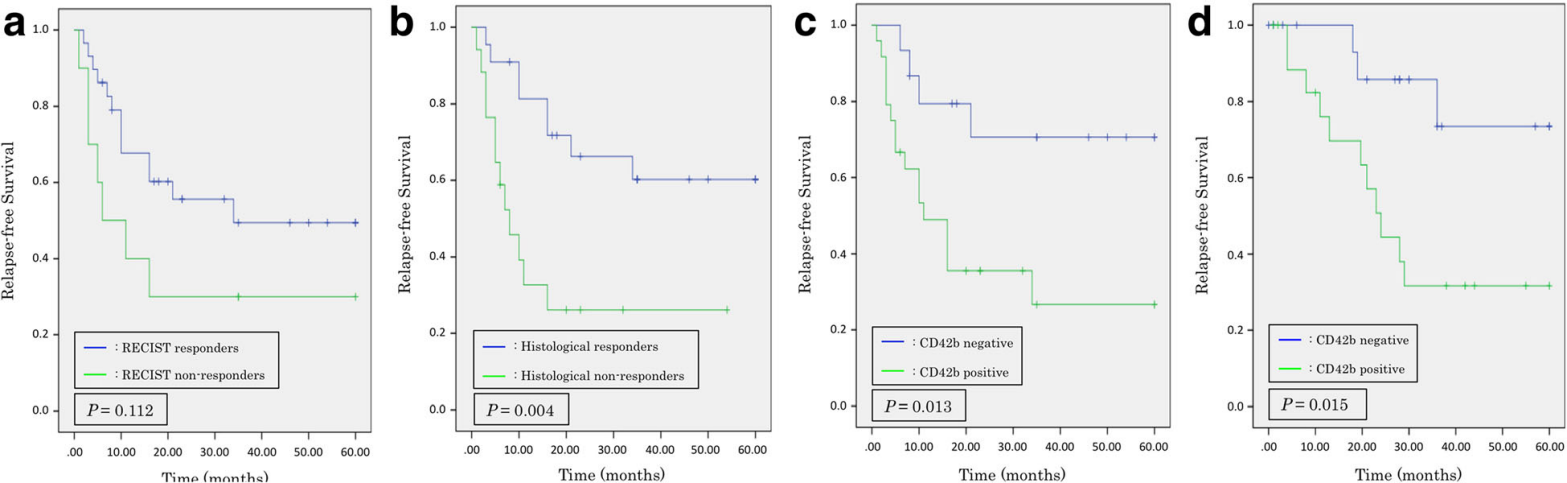
Relapse-free survival curves for responders and non-responders in the p-DCS group and CD42b expression in the both groups. **a**: RECIST responders (*P* = 0.112; log-rank test). **b**: Histological responders (*P* = 0.004; log-rank test). **c**: CD42b expression in the p-DCS group (*P* = 0.013; log-rank test). **d**: CD42b expression in the control group (*P* = 0.015; log-rank test). *DCS,* docetaxel, cisplatin, and S-1; *RECIST,* Response Evaluation Criteria in Solid Tumors

Relapse-free survival curves for the patients in the both groups are shown in Fig. 3. In the p-DCS group, there was no significant difference in prognosis between the RECIST responders and non-responders (*P* = 0.112) (Fig. 3a). Histological evaluation and CD42b expression showed that relapse-free survival was significantly longer in responders than non-responders (*P* = 0.004, *P* = 0.013, respectively) (Fig. 3b, c). In the control group, the relapse-free survival was significantly longer in CD42b-negative than in CD42b-positive patients (*P* = 0.015) (Fig. 3d).

In the p-DCS group, univariate analysis showed that histological findings (*P* = 0.023) and CD42b expression (*P* = 0.021) were significantly associated with OS. The four variables (sex, hepatic metastasis, histological evaluation, and CD42b expression) that were found to be significant by univariate analysis and therefore had prognostic potential (*P* < 0.10) were subjected to multivariate analysis. Multivariate analysis identified that male sex (hazard ratio: 0.281, 95% confidence interval: 0.093–0.846, *P* = 0.024) was correlated with good prognosis and CD42b expression (hazard ratio: 4.406, 95% confidence interval: 1.325–14.65, *P* = 0.016) with poor prognosis (Table 5).

**Table 5:** Univariate/multivariate analyses of factors associated with prognosis in the p-DCS group

		Univariate analysis				Multivariate analysis		
Variable		No. of patients	HR	95% CI	*P* value	HR	95% CI	*P* value
Age (years)	≥70	15	1.470	(0.607–3.560)	0.393			
	<70	24						
Gender	Male	32	0.409	(0.156–1.075)	0.070	0.281	(0.093–0.846)	0.024
	Female	7						
ECOG performance status	≥1	2	0.894	(0.119–6.698)	0.913			
	0	37						
Borrmann macroscopic type	Non-type 4	38	0.452	(0.059–3.439)	0.443			
	Type 4	1						
Differentiation	Diffuse	18	0.758	(0.310–1.854)	0.543			
	Intestinal	21						
PAN metastasis	(+)	16	1.869	(0.539–4.854)	0.201			
	(−)	23						
Hepatic metastasis	(+)	9	2.508	(0.993–6.333)	0.052	1.718	(0.530–5.570)	0.367
	(−)	30						
RECIST	SD, PD	10	1.769	(0.705–4.439)	0.225			
	CR, PR	29						
Histological evaluation	0, 1a, 1b	17	2.84	(1.152–7.000)	0.023	1.938	(0.612–6.129)	0.260
	2, 3	22						
CD42b expression	≥10%	24	3.644	(1.213–10.95)	0.021	4.406	(1.325–14.65)	0.016
	<10%	15						
Podoplanin expression	(+)	28	1.411	(0.512–3.889)	0.505			
	(−)	11						
SNAIL expression	(+)	30	1.736	(0.664–4.539)	0.261			
	(−)	9						
FOXP3 expression	(+)	7	1.272	(0.369–4.386)	0.703			
	(−)	32						

*CI* confidence interval, *CR* complete response, *ECOG* Eastern Cooperative Oncology Group, *FOXP3* forkhead box P3, *HR* hazard ratio, *PD* progressive disease, *PR* partial response, *RECIST* Response Evaluation Criteria in Solid Tumors, *SD* stable diseases

## Discussion

S-1 is a standard postoperative chemotherapy for patients who have undergone curative gastrectomy and D2 lymphadenectomy for locally advanced gastric cancer in Japan [20]. DCS therapy has been found to be effective in several trials [26–28] and is expected to become the next standard regimen for advanced gastric cancer in Japan because it results in a sufficient R0 resection rate and good histological response rate. According to multivariate analysis, expression of CD42b, a platelet marker, in our biopsy specimens from advanced gastric cancer with preoperative DCS therapy was significantly associated with chemoresistance. In the p-DCS group, the prognosis was significantly longer in the CD42b-negative than the CD42b-positive patients and histological responders had significantly longer survival than the non-responders. According to multivariate analysis, male sex and CD42b expression were significantly associated with OS. Similarly, in the control group, the OS was significantly longer in CD42b-negative than in CD42b-positive patients.

In the p-DCS group, the reasons for a significantly association between male sex and better prognosis remain uncertain. However, one possible reason is that our findings were affected by the numbers of male (32) and female (seven) patients. Also, 13/32 (41%) men had died, compared with 6/7 (86%) women. The female mortality rate (86%) may have influenced the association between male sex and better prognosis. Although there was a significant association between male sex and OS in this study, it was considered of no particular importance.

Although Takahari et al. [29] have proposed a novel prognostic index consisting of four factors (performance status ≥1, ≥two metastatic sites, no prior gastrectomy, and high serum alkaline phosphatase concentration), this index was considered unsuitable for our cases (data not shown).

It has been suggested that platelets are one of the factors promoting cancer migration, infiltration, and metastasis [30]. Although intravasated platelet aggregation has focused attention on EMT, EPA has been less noticeable. Hematoxylin and eosin staining cannot be used to confirm the presence of EPA in cancer stroma because platelets lack nuclei. EPA signifies platelet aggregation in the extravascular space, in which there are usually no platelets, and these platelets release microparticles into the surrounding environment. Platelets contain high concentrations of TGF-β, which is secreted by activated platelets [31, 32]. TGF-β enhances invasion, metastasis, and chemoresistance in cancer stroma through induction of EMT [32]. One study has suggested that the EMT marker SNAIL is associated with chemoresistance [17] and we found a significant relationship between SNAIL expression and chemoresistance in our study. However, we did not find a significant relationship between CD 42b expression and SNAIL expression. A possible explanation for the lack of correlation between SNAIL expression and CD42b expression is that many factors can induce SNAIL expression in cancer microenvironments. Not only TGF-β signal but also other signaling pathways such as Notch, Wnt, Hedgehog, AKT-mTOR, MAPK/ERK, and NF-kB pathways can induce SNAIL expression [33]. This may explain why we found no correlation between SNAIL expression and CD42b expression.

Oshimori et al. [34] have reported that the distribution of TGF-β coincides with vasculature and monocytic myeloid cells in tumor microenvironments and that TGF-β signaling is at the root of cancer heterogeneity. The heterogeneity of cancer cells is also related to chemoresistance, distant metastasis, malignant transformation, and cancer recurrence. Our findings suggest that the presence of EPA in the cancer microenvironment induces a concentration gradient of TGF-β, resulting in heterogeneity of cancer and stromal cells.

TGF-β also enhances induction of immune tolerance by Treg cell infiltration into cancer stroma, which contributes to chemoresistance [35]. TGF-β-induced FOXP3^+^ Treg cells participate in maintenance of immunosuppression [36, 37] and play critical roles in chemoresistance [35]. Myeloid-derived suppressor cells (MDSCs) may mediate the development of Treg cells through a combination of pathways dependent on TGF-β [38–40]. Expression of the Treg cell marker FOXP3 contributes to immune tolerance [33, 34] and chemoresistance [35]; however, we found no relationship between FOXP3 expression and chemoresistance in our study.

Because there is a close relationship between MDSCs and Treg cell induction, when MDSCs are blocked by docetaxel [41] and 5-fluorouracil [42], the number and function of Treg cells decrease and anti-tumor immune responses recover. This explains why expression of the Treg cell marker FOXP3 was not associated with chemoresistance in our study.

This study had some limitations. First, histological evaluation is more subjective than RECIST; therefore, there may have been some issues with inter-rater reliability. Evaluation of residual tumor volume may vary between pathologists because there is no consensus on a morphological definition of viable cancer cells. Moreover, in poorly differentiated adenocarcinomas the interface between tumor and stroma is unclear because of poor formation of the ducts and alveolar structures and fibrosis of stroma. Second, there is a concern about heterogeneity of tumor characteristics. In an attempt to minimize the effects of histological heterogeneity of our patients’ gastric cancers, we performed as evaluated expression of CD42b in available resected specimens and biopsies. Third, this study enrolled the patients who had received preoperative DCS therapy and postoperative chemotherapy of S-1 alone. Future studies should evaluate CD42b expression in patients undergoing standard regimen such as S-1 plus cisplatin or the few available second-line therapies. Fourth, our study was small, retrospective, and conducted in a single institution; therefore, further larger, multi-center studies are required to validate our results.

## Conclusions

Our findings indicate that EPA in gastric cancer biopsy specimens is associated with OS, suggesting that EPA could become a new prognostic factor for OS. Moreover, EPA could be a predictor of response to both preoperative and postoperative setting and could therefore be used to guide changes in dosage or other regimens. CD42b immunohistochemistry may be useful not only for preoperative or postoperative chemotherapy but also for chemotherapy for unresectable recurrent gastric cancer. Further studies are needed to investigate the relationship between CD42b expression and unresectable recurrent gastric cancer. We believe our study is the first report of an association between EPA and prognosis of advanced gastric cancer.

## Abbreviations

CAF: Cancer-associated fibroblast
CR: Complete response
CT: Computed tomography
DCS: Docetaxel, cisplatin, and S-1
ECOG: Eastern Cooperative Oncology Group
EGD: Esophagogastroduodenoscopy
EMT: Epithelial–mesenchymal transition
EPA: Extravasated platelet aggregation
FOXP3: Forkhead box P3
HR: Hazard ratio
IRS: Immunoreactivity score
JCGC: Japanese Classification of Gastric Carcinoma
MDSCs: Myeloid-derived suppressor cells
OR: Odds ratio
OS: Overall survival
PAN: Para-aortic lymph node
PAND: Para-aortic lymph node dissection
PD: Progressive disease
p-DCS: Preoperative DCS therapy
PP: Podoplanin-positive
PR: Partial response
RECIST: Response Evaluation Criteria in Solid Tumors
SD: Stable disease
SI: Staining intensity
TGF-β: Transforming growth factor-β
Treg cell: Regulatory T cell

## References

[1] Torre LA, Bray F, Siegel RL, Ferlay J, Lortet-Tieulent J, Jemal A (2015). Global cancer statistics, 2012. CA Cancer J Clin.

[2] Sakuramoto S, Sasako M, Yamaguchi T, Kinoshita T, Fujii M, Nashimoto A, Furukawa H, Nakajima T, Ohashi Y, Imamura H (2007). Adjuvant chemotherapy for gastric cancer with S-1, an oral fluoropyrimidine. N Engl J Med.

[3] Fushida S, Fujimura T, Oyama K, Yagi Y, Kinoshita J, Ohta T (2009). Feasibility and efficacy of preoperative chemotherapy with docetaxel, cisplatin and S-1 in gastric cancer patients with para-aortic lymph node metastases. Anti-Cancer Drugs.

[4] Oyama K, Fushida S, Kinoshita J, Makino I, Nakamura K, Hayashi H, Nakagawara H, Tajima H, Fujita H, Takamura H (2012). Efficacy of pre-operative chemotherapy with docetaxel, cisplatin, and S-1 (DCS therapy) and curative resection for gastric cancer with pathologically positive para-aortic lymph nodes. J Surg Oncol.

[5] Kinoshita J, Fushida S, Tsukada T, Oyama K, Okamoto K, Makino I, Nakamura K, Miyashita T, Tajima H, Takamura H (2015). Efficacy of conversion gastrectomy following docetaxel, cisplatin, and S-1 therapy in potentially resectable stage IV gastric cancer. Eur J Surg Oncol.

[6] Tsuburaya A, Mizusawa J, Tanaka Y, Fukushima N, Nashimoto A, Sasako M (2014). Neoadjuvant chemotherapy with S-1 and cisplatin followed by D2 gastrectomy with para-aortic lymph node dissection for gastric cancer with extensive lymph node metastasis. The British journal of surgery.

[7] Wang Y, Yu YY, Li W, Feng Y, Hou J, Ji Y, Sun YH, Shen KT, Shen ZB, Qin XY (2014). A phase II trial of Xeloda and oxaliplatin (XELOX) neo-adjuvant chemotherapy followed by surgery for advanced gastric cancer patients with para-aortic lymph node metastasis. Cancer Chemother Pharmacol.

[8] Eisenhauer EA, Therasse P, Bogaerts J, Schwartz LH, Sargent D, Ford R, Dancey J, Arbuck S, Gwyther S, Mooney M (2009). New response evaluation criteria in solid tumours: revised RECIST guideline (version 1.1). Eur J Cancer.

[9] Kurokawa Y, Shibata T, Ando N, Seki S, Mukaida H, Fukuda H (2013). Which is the optimal response criteria for evaluating preoperative treatment in esophageal cancer: RECIST or histology?. Ann Surg Oncol.

[10] Kurokawa Y, Shibata T, Sasako M, Sano T, Tsuburaya A, Iwasaki Y, Fukuda H (2014). Validity of response assessment criteria in neoadjuvant chemotherapy for gastric cancer (JCOG0507-a). Gastric Cancer.

[11] Bambace NM, Holmes CE (2011). The platelet contribution to cancer progression. Journal of thrombosis and haemostasis: JTH.

[12] Tsuruo T, Fujita N (2008). Platelet aggregation in the formation of tumor metastasis. Proceedings of the Japan Academy Series B, Physical and biological sciences.

[13] Qi C, Li B, Guo S, Wei B, Shao C, Li J, Yang Y, Zhang Q, Li J, He X (2015). P-selectin-mediated adhesion between platelets and tumor cells promotes Intestinal tumorigenesis in Apc(min/+) mice. Int J Biol Sci.

[14] Mikami J, Kurokawa Y, Takahashi T, Miyazaki Y, Yamasaki M, Miyata H, Nakajima K, Takiguchi S, Mori M, Doki Y (2016). Antitumor effect of antiplatelet agents in gastric cancer cells: an in vivo and in vitro study. Gastric Cancer.

[15] Miyashita T, Tajima H, Makino I, Nakagawara H, Kitagawa H, Fushida S, Harmon JW, Ohta T (2015). Metastasis-promoting role of extravasated platelet activation in tumor. J Surg Res.

[16] Iwatsuki M, Mimori K, Yokobori T, Ishi H, Beppu T, Nakamori S, Baba H, Mori M (2010). Epithelial-mesenchymal transition in cancer development and its clinical significance. Cancer Sci.

[17] Foroni C, Broggini M, Generali D, Damia G (2012). Epithelial-mesenchymal transition and breast cancer: role, molecular mechanisms and clinical impact. Cancer Treat Rev.

[18] Yoshida K, Yamaguchi K, Okumura N, Tanahashi T, Kodera Y (2016). Is conversion therapy possible in stage IV gastric cancer: the proposal of new biological categories of classification. Gastric Cancer.

[19] Japanese classification of gastric carcinoma (2011). 3rd English edition. Gastric Cancer.

[20] Sasako M, Sakuramoto S, Katai H, Kinoshita T, Furukawa H, Yamaguchi T, Nashimoto A, Fujii M, Nakajima T, Ohashi Y (2011). Five-year outcomes of a randomized phase III trial comparing adjuvant chemotherapy with S-1 versus surgery alone in stage II or III gastric cancer. J Clin Oncol.

[21] Becker K, Mueller JD, Schulmacher C, Ott K, Fink U, Busch R, Bottcher K, Siewert JR, Hofler H (2003). Histomorphology and grading of regression in gastric carcinoma treated with neoadjuvant chemotherapy. Cancer.

[22] Ishikawa S, Miyashita T, Inokuchi M, Hayashi H, Oyama K, Tajima H, Takamura H, Ninomiya I, Ahmed AK, Harman JW (2016). Platelets surrounding primary tumor cells are related to chemoresistance. Oncol Rep.

[23] Tong L, Yuan S, Feng F, Zhang H (2012). Role of podoplanin expression in esophageal squamous cell carcinoma: a retrospective study. Dis Esophagus.

[24] Keck B, Wach S, Goebell PJ, Kunath F, Bertz S, Lehmann J, Stockle M, Taubert H, Wullich B, Hartmann A (2013). SNAI1 protein expression is an independent negative prognosticator in muscle-invasive bladder cancer. Ann Surg Oncol.

[25] Oda N, Shimazu K, Naoi Y, Morimoto K, Shimomura A, Shimoda M, Kagara N, Maruyama N, Kim SJ, Noguchi S (2012). Intratumoral regulatory T cells as an independent predictive factor for pathological complete response to neoadjuvant paclitaxel followed by 5-FU/epirubicin/cyclophosphamide in breast cancer patients. Breast Cancer Res Treat.

[26] Nakayama N, Koizumi W, Sasaki T, Higuchi K, Tanabe S, Nishimura K, Katada C, Nakatani K, Takagi S, Saigenji K (2008). A multicenter, phase I dose-escalating study of docetaxel, cisplatin and S-1 for advanced gastric cancer (KDOG0601). Oncology.

[27] Sato Y, Takayama T, Sagawa T, Takahashi Y, Ohnuma H, Okubo S, Shintani N, Tanaka S, Kida M, Sato Y (2010). Phase II study of S-1, docetaxel and cisplatin combination chemotherapy in patients with unresectable metastatic gastric cancer. Cancer Chemother Pharmacol.

[28] Hirakawa M, Sato Y, Ohnuma H, Takayama T, Sagawa T, Nobuoka T, Harada K, Miyamoto H, Sato Y, Takahashi Y (2013). A phase II study of neoadjuvant combination chemotherapy with docetaxel, cisplatin, and S-1 for locally advanced resectable gastric cancer: nucleotide excision repair (NER) as potential chemoresistance marker. Cancer Chemother Pharmacol.

[29] Takahari D, Boku N, Mizusawa J, Takashima A, Yamada Y, Yoshino T, Yamazaki K, Koizumi W, Fukase K, Yamaguchi K (2014). Determination of prognostic factors in Japanese patients with advanced gastric cancer using the data from a randomized controlled trial, Japan clinical oncology group 9912. Oncologist.

[30] Lowe KL, Navarro-Nunez L, Watson SP (2012). Platelet CLEC-2 and podoplanin in cancer metastasis. Thromb Res.

[31] Assoian RK, Komoriya A, Meyers CA, Miller DM, Sporn MB (1983). Transforming growth factor-beta in human platelets. Identification of a major storage site, purification, and characterization. J Biol Chem.

[32] Labelle M, Begum S, Hynes RO (2011). Direct signaling between platelets and cancer cells induces an epithelial-mesenchymal-like transition and promotes metastasis. Cancer Cell.

[33] Du B, Shim JS. Targeting Epithelial-Mesenchymal Transition (EMT) to Overcome Drug Resistance in Cancer. Molecules (Basel, Switzerland). 2016;21(7):965–79. 10.3390/molecules21070965 PMC6273543 27455225

[34] Oshimori N, Oristian D, Fuchs E (2015). TGF-beta promotes heterogeneity and drug resistance in squamous cell carcinoma. Cell.

[35] Liu H, Zhang T, Ye J, Li H, Huang J, Li X, Wu B, Huang X, Hou J (2012). Tumor-infiltrating lymphocytes predict response to chemotherapy in patients with advance non-small cell lung cancer. Cancer immunology, immunotherapy: CII.

[36] Nishikawa H, Sakaguchi S (2010). Regulatory T cells in tumor immunity. Int J Cancer.

[37] Winkler I, Wilczynska B, Bojarska-Junak A, Gogacz M, Adamiak A, Postawski K, Darmochwal-Kolarz D, Rechberger T, Tabarkiewicz J (2015). Regulatory T lymphocytes and transforming growth factor beta in epithelial ovarian tumors-prognostic significance. J Ovarian Res.

[38] Huang B, Pan PY, Li Q, Sato AI, Levy DE, Bromberg J, Divino CM, Chen SH (2006). Gr-1+CD115+ immature myeloid suppressor cells mediate the development of tumor-induced T regulatory cells and T-cell anergy in tumor-bearing host. Cancer Res.

[39] Diaz-Montero CM, Finke J, Montero AJ (2014). Myeloid-derived suppressor cells in cancer: therapeutic, predictive, and prognostic implications. Semin Oncol.

[40] Serafini P, Mgebroff S, Noonan K, Borrello I (2008). Myeloid-derived suppressor cells promote cross-tolerance in B-cell lymphoma by expanding regulatory T cells. Cancer Res.

[41] Kodumudi KN, Woan K, Gilvary DL, Sahakian E, Wei S, Djeu JY (2010). A novel chemoimmunomodulating property of docetaxel: suppression of myeloid-derived suppressor cells in tumor bearers. Clin Cancer Res.

[42] Vincent J, Mignot G, Chalmin F, Ladoire S, Bruchard M, Chevriaux A, Martin F, Apetoh L, Rebe C, Ghiringhelli F (2010). 5-fluorouracil selectively kills tumor-associated myeloid-derived suppressor cells resulting in enhanced T cell-dependent antitumor immunity. Cancer Res.

